# Prevalence and Factors Associated With Severity of Undernutrition Among Children Aged 6–59 Months in Tanzania

**DOI:** 10.1002/fsn3.4648

**Published:** 2024-12-10

**Authors:** Ahmed Gharib Khamis, Akwilina Wendelin Mwanri, Julius Edward Ntwenya

**Affiliations:** ^1^ Zanzibar Agricultural and Livestock Research Institute Zanzibar Tanzania; ^2^ Department of Human Nutrition and Consumer Sciences Sokoine University of Agriculture Morogoro Tanzania; ^3^ Department of Public Health and Community Nursing The University of Dodoma Dodoma Tanzania

**Keywords:** anemia, children, severe undernutrition, stunting, Tanzania, wasting

## Abstract

Undernutrition has been identified as a significant public health challenge in developing nations like Tanzania. Severe childhood undernutrition is a life‐threatening problem that can result in impaired growth, weakened immune systems, and even death. The primary aim of this study was to identify the prevalence of multiple forms of severe undernutrition and factors associated with the severity of childhood undernutrition in children aged 6–59 months in Tanzania. This was a secondary data analysis conducted on a cross‐sectional survey obtained from the 2022 Tanzania Demographic and Health Survey Malaria Indicator Survey (TDHS‐MIS). This analysis comprised 4224 children with complete information. The study employed the proportional odds model of ordinal logistic regression to identify factors associated with severity of stunting, wasting, and anemia. Out of the 4224 included children, 8.6% were severely stunted, 0.8% were severely wasted, and 1.7% were severely anemic. The prevalence of multiple forms of severe undernutrition was 10.4%. Significant associations were observed between socioeconomic factors—including the gender and age of the household head, household wealth status, possession of health insurance, and availability of mosquito nets with severe undernutrition. Maternal factors such as nutrition status, current age, age at first birth, education, marriage, and occupation were related to the severity of undernutrition. Approximately one children out of every 10 suffers from some form of severe undernutrition in Tanzania. Several factors including maternal and socioeconomic were identified as potentially influencing severe undernutrition. Multifaceted efforts are required to lessen the severity of undernutrition.

## Graphical abstract

The aim of this study was to identify the prevalence of multiple forms of severe undernutrition and factors associated with severity of childhood undernutrition in children aged 6–59 months in Tanzania. The prevalence of multiple forms of severe undernutrition was 10.4%. Severe undernutrition was associated with the gender of children, age of the household head, wealth status, possession of health insurance, and availability of mosquito nets, as well as maternal factors such as nutrition status, current age, age at first birth, education, marriage, and occupation.

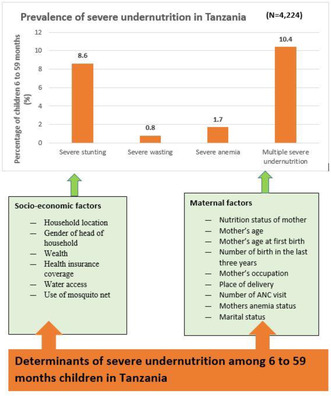

## 1 Introduction

Undernutrition remains a significant public health challenge globally, particularly for children the age of five in low‐ and middle‐income countries (LMICs) (Obasohan et al. 2020; Govender et al. 2021). Globally, in 2022, 2.5 billion adults were overweight, including 890 million who were living with obesity, while 390 million were underweight. Also, 149 million children under 5 years were estimated to be stunted (too short for age), 45 million were estimated to be wasted (too thin for height), and 37 million were overweight or living with obesity (World Health Organization 2022). It was estimated that undernutrition is associated with 2.7 million child deaths annually, which is approximately equal to 45% of all child deaths (Infant and young child feeding n.d.).

Despite the appreciable progress of Tanzania, it still faces challenges related to severe undernutrition, which can have negative and long‐term consequences for a child's overall health, growth, and development (Beckstead et al. 2022). Most Tanzanians depend on agriculture for their livelihoods. The country had a significant decline in the poverty level from 34% in 2007 to 26% in 2018 and was officially included among lower middle‐income countries (World Bank n.d.). Additionally, a significant share of the population is food‐insecure with a large proportion of malnutrition, especially children under 5 years of age and women of reproductive age. For example, data showed that about 30% of all under‐five children in the country are stunted and 9% are severely stunted (Ministry of Health CD, Gender and Elderly, Ministry of Health (Zanzibar), National Bureau of Statistics, OCGS, and ICF 2015). Also, about 3.0% of the children are wasted and 12% are underweight (Ministry of Health CD, Gender and Elderly, Ministry of Health (Zanzibar), National Bureau of Statistics, OCGS, and ICF 2015). Only 19% of the children between 6 and 23 months can afford to obtain a minimum dietary diversity, while 7% and 30% consume unhealthy food and sweet beverages, respectively (Ministry of Health CD 2018). Overall, the prevalence of anemia was 59%; a higher prevalence was found among those of aged 6–23 months (73%) compared to those aged 24–59 months (52%) (Ministry of Health, Community Development, Gender, Elderly and Children (MoHCDGEC) [Tanzania Mainland], Ministry of Health (MoH) [Zanzibar], National Bureau of Statistics (NBS), Office of the Chief Government Statistician (OCGS), and ICF 2016). There is a notable regional variation in the prevalence of undernutrition, with regions that have high food production experiencing persistently high prevalence of chronic undernutrition (stunting) (Ministry of Health, Community Development, Gender, Elderly and Children (MoHCDGEC) [Tanzania Mainland], Ministry of Health (MoH) [Zanzibar], National Bureau of Statistics(NBS), Office of the Chief Government Statistician (OCGS), and ICF 2016). This regional disparity in stunting prevalence could be influenced by various factors such as socioeconomics, access to nutritious foods, healthcare services, and sanitation facilities (Sunguya et al. 2019).

Previous studies have indicated several factors, such as socioeconomic, dietary, and maternal factors, determine the severity and moderate levels of undernutrition in Tanzania and outside (Arimond and Ruel 2004; Motbainor, Worku, and Kumie 2015; Ahmad, Khalique, and Khalil 2018; Khamis et al. 2019). However, evidence on the prevalence and factors determining severe forms of undernutrition are scarce (Chirande et al. 2015). Understanding the magnitude of severe forms of undernutrition and its determinants could help inform health and nutrition policies that focus on enhancing the health services and quality of child nutrition. Factors such as child feeding, wealth, and maternal nutritional status have been reported in many studies as contributors to undernutrition (Sunguya et al. 2019).

Severe undernutrition can have long‐term and negative consequences on a child's physical and mental growth, causing increasing illness and death rates, as well as a decreased potential for future achievements (De Sanctis et al. 2021). Further consequences of severe undernutrition in early childhood are on their cognitive development, educational attainment, and economic productivity in later life (Suryawan et al. 2022). By examining a wide range of factors, we seek to identify the most significant determinants of severe undernutrition in this population. The results of this analysis will contribute to the existing body of knowledge on child nutrition in Tanzania and provide evidence‐based recommendations for interventions aimed at reducing the burden of undernutrition in this vulnerable population.

## 2 Materials and Methods

### 2.1 Data Source

This study is based on secondary data from a cross‐sectional survey of the 2022 Tanzania Demographic and Health Survey Malaria Indicator Survey (TDHS‐MIS). This survey was coordinated by the National Bureau of Statistics (NBS) in Tanzania Mainland and the Office of the Chief Government Statistician (OCGS) in Zanzibar (Ministry of Health, Community Development, Gender, Elderly and Children (MoHCDGEC) [Tanzania Mainland], Ministry of Health (MoH) [Zanzibar], National Bureau of Statistics(NBS), Office of the Chief Government Statistician (OCGS), and ICF 2016). All procedures concerning the data collection are available in the TDHS‐MIS 2022 final report (Ministry of Health, Community Development, Gender, Elderly and Children (MoHCDGEC) [Tanzania Mainland], Ministry of Health (MoH) [Zanzibar], National Bureau of Statistics(NBS), Office of the Chief Government Statistician (OCGS), and ICF 2016). This survey was purposely designed to provide representative results at the country, regional, and rural–urban levels. This survey is part of the global program to determine demographic and health information, which aims to facilitate countries to obtain accurate data as well as monitor and evaluate the health and nutrition of a population. This national survey involved a two‐stage sampling design. Firstly, 629 clusters (sample points) were selected randomly. Secondly, 26 households from the selected clusters were selected. A total sample of 16,354 households were included in the survey. All data were extracted from an online database to get information about socioeconomic, maternal, and nutrition status and the dietary intake of children (Rasheed and Jeyakumar 2018). From the database, 4224 children aged 6–59 months living with their parents or guardian were selected in the analysis. Details of the procedures and steps used to select children can be found in a previous study (Khamis et al. 2019).

### 2.2 Anthropometric Measurements

Anthropometric measurements, including weight and height, were done in accordance with the protocol from the World Health Organization by directly measuring children's weight and height (WHO: Physical status and the use and interpretation of anthropometry 2006). The outcome variables of interest, such as severe stunting, wasting, and underweight, were calculated by using the *Z*‐scores of height‐for‐age, weight‐for‐height, and weight‐for‐age, based on 2006 WHO child growth standards (WHO: Physical status and the use and interpretation of anthropometry 2006). Height‐for‐age *z*‐scores are for measuring chronic undernutrition, which may be due to prolonged food deprivation. Children who are below minus three standard deviations (−3SD) from the were considered severely stunted and wasted, respectively (WHO: Physical status and the use and interpretation of anthropometry 2006). In addition, the magnitude of anemia was measured by using hemoglobin concentration (< 11.0 g/dL), and the cutoff point for severe anemia was < 8.0 g/dL. Multiple forms of severe undernutrition were calculated if a child was having severe stunted, severe wasted, or severe anemia.

### 2.3 Assessment of Other Factors

This study adopted socioeconomic and maternal factors as suggested by previous studies and the malnutrition framework (Sunguya et al. 2019). Covariates measured included demographic characteristics of children and household as well as age and gender of the children. Children's ages were categorized into two ranges: (1) between 6 and 23 months, and (2) between 24 and 59 months. Further covariates included the household location (rural or urban) and marital status: married, not married, or divorced. Other reported characteristics from the mother were the place of delivery, which can be at home, at a health facility, or at other places. Other maternal information were recorded such as age at first delivery, educational attainment, occupation, and number of children born in the previous five 5 years. The categorization of this information was based on previous studies (Sunguya et al. 2019; Chirande et al. 2015). Furthermore, nutritional status of the mother was determined using the body mass index (BMI) and anemia status of the mother. Other socioeconomic information included were gender and age of the household head, which was separated into three categories: (1) 15–29 years, 30–49 years, and over ≥ 50 years. The household wealth index was derived from principal component analysis (PCA), and it was already divided into five quintiles of poorest, poorer, middle, richer, and richest (Ministry of Health CD 2018; Ministry of Health, Community Development, Gender, Elderly and Children (MoHCDGEC) [Tanzania Mainland], Ministry of Health (MoH) [Zanzibar], National Bureau of Statistics(NBS), Office of the Chief Government Statistician (OCGS), and ICF 2016; Sunguya et al. 2019).

### 2.4 Conceptual Framework

**Figure:**
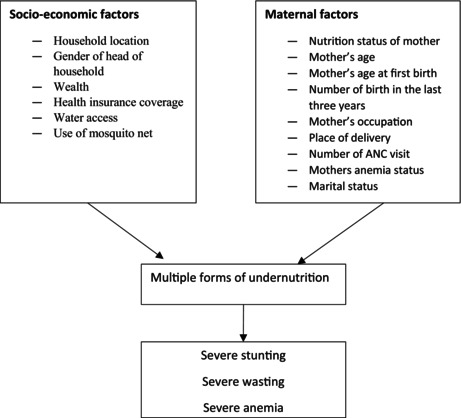
Adopted from UNICEF Malnutrition Framework, 2020

#### 2.4.1 Statistical Analysis

All descriptive statistics were utilized to depict the findings with frequency and proportions for categorical variables, and for other continuous variables, means with standard deviations (Means ± SD) were used. We conducted multivariate ordinal logistic regression analyses by employing a proportional odds model (POM) to explore the association among maternal, socioeconomic, and other factors and the severity of undernutrition indicators, including stunting, wasting, and anemia. We considered the ordinal nature of the outcome variable in our analysis (Das and Rahman 2011). Statistical significance was determined at *p* < 0.05. The analyses were carried out using Statistical Package for the Social Science (SPSS) software version 23, with confirmation in Stata software.

## 3 Results

### 3.1 Descriptive Characteristics

Table 1 shows the descriptive characteristics of 4224 children enrolled in this study. An almost equal number of male and female children were involved in the study. About 65% of them were of age between 24 and 59 months, and about one‐third (35%) were less than 24 months. Most of them were born at the health facilities (82.3%) compared to other places.

**TABLE 1:** Descriptive characteristics of the included participants (*N* = 4224).

Variables		Frequency	Percentage
Socioeconomic			
Residence	Urban	1148	27.2
	Rural	3076	72.8
Gender of head of the household	Male	3271	77.4
	Female	953	22.6
Age of the household head (years)	15–29	744	17.6
	30–49	2474	58.6
	Above 50	1006	23.8
Household wealth	Poorest	903	21.4
	Poorer	826	19.6
	Middle	869	20.6
	Richer	867	20.5
	Richest	759	18.0
Health insurance coverage	No	4043	95.7
	Yes	181	4.3
Water sources	Protected	2975	70.4
	Unprotected	1246	29.5
Presence of mosquito net	No	736	17.4
	Yes	3488	82.6
Maternal charactetistics			
Nutrition status of mother (BMI)	Underweight	299	7.1
	Normal	2671	63.2
	Overweight	788	18.7
	Obesity	466	11.0
Age of mother (years)	15–24	1098	26.0
	25–39	2652	62.8
	40–49	474	11.2
Age of mother during first birth (years)	10–24	2909	68.9
	25–49	1315	31.1
Number of births in the last 3 years	None	803	19.0
	One	2876	68.1
	Two	528	12.5
	Three	17	0.4
Mother's occupation	Not working	1425	33.7
	Farmer	938	22.2
	Employed	1688	40.0
Education of mother	Others	173	4.1
	No education	911	21.6
	Primary	2244	53.1
	Secondary	1029	24.4
	Higher	40	1.0
Place of birth	At health facility	2025	82.3
	At home	435	17.7
	Other places	2	0.1
Number of antenatal care visits	No	259	6.1
	1–5	1424	33.7
	≥ 6	2541	60.2
Anemia status of mother	No	2521	59.9
	Mild	828	19.7
	Moderate	772	18.3
	Severe	90	2.1
	Not measured	13	
Current marital status	No	634	15.0
	Yes	3590	85.0
Child characteristics and severity of malnutrition			
Gender of children	Male	2135	50.5
	Female	2089	49.5
Age of children (months)	6–23	1473	34.9
	24–59	2751	65.1
Dietary diversity	Yes (≥ 5 food groups)	230	5.5
	No	3994	94.6

This analysis reveals that the majority of households included in the study were situated in rural areas (72.8%), with males predominantly serving as heads of these households (77.4%). Regarding wealth, the richest households accounted for 18%, while the poorest represented 21.4%. Furthermore, a significant portion of mothers were engaged in employment (40%), with the majority of the participants having attained the primary education level (53.1%). Concerning dietary habits, a high proportion of children (94.6%) did not meet the recommended dietary intake of five food groups in the 24 h preceding the survey. Only a few of them (5.5%) had achieved dietary diversification. The most commonly consumed food groups were grains, roots, and tubers (50.7%), while a lower proportion of children reported consumption of animal‐sourced foods such as eggs (2.4%) and milk or dairy products (7.5%).

### 3.2 Prevalence of Severe Undernutrition

Table 2 shows the prevalence of moderate to severe undernutrition. The prevalence of stunting (chronic malnutrition) was 21.8%, and for severe stunting, it was was 8.6%. Only 3.0% had wasting (acute malnutrition), while less than 1% had severe wasting. About 31.7% were moderately anemic, followed by mild anemia (27.1%) and severe anemia (1.7%). The prevalence of multiple forms of severe undernutrition was estimated to be 10.4%.

**TABLE 2:** Prevalence of severity of undernutrition.

Variable		Frequency	Percentage
Stunting	No	2939	69.6
	Moderate	920	21.8
	Severe	365	8.6
Wasting	No	4066	96.3
	Moderate	126	3.0
	Severe	32	0.8
Anemia	No	1663	39.5
	Mild	1142	27.1
	Moderate	1335	31.7
	Severe	71	1.7
Multiple forms of severe undernutrition	No	3783	89.5
	Yes	441	10.44

### 3.3 Socioeconomic Factors and Severity of Undernutrition

In Table 3, the findings regarding the association between socioeconomic factors and the severity of undernutrition are presented. This shows that the gender and age of the household head, household wealth status, health insurance coverage, and availability of mosquito nets were significantly related to the severity of stunting, wasting, and anemia. Children in households headed by females had a lower likelihood of severe stunting compared to those in male‐headed households (AOR = 0.82, 95% CI: 0.70–0.97). Also, the severity of stunting and anemia decreased among under‐five children whose parents were aged between 30 and 49 years compared to those aged between 15 and 29 years. The severity of stunting and wasting decreased with the increase in wealth status of the households. Children from the richest households were less likely to experience stunting compared to those from the poorest families (AOR = 0.26, 95% CI: 0.20–0.36). Additionally, the coverage of health insurance within the household was significantly associated with a reduction in the severity of anemia (AOR = 0.53, 95% CI: 0.39–0.71). Similarly, the presence of mosquito nets in the households was associated with a decrease in the severity of stunting (AOR = 0.79, 95% CI: 0.67–0.94).

**TABLE 3:** Ordinal logistic regression analysis between socioeconomic factors on the severity of malnutrition among children aged 6–59 months in Tanzania.

Variables	Stunting		Wasting		Anemia	
	AOR (95% CI)	*p*	AOR (95% CI)	*p*	AOR (95% CI)	*p*
Place of residence						
Urban	Ref		Ref		Ref	
Rural	0.99 (0.81–1.21)	0.952	1.11 (0.70–1.76)	0.641	0.96 (0.82–1.13)	0.683
Gender of head of the household						
Male	Ref		Ref		Ref	
Female	0.82 (0.70–0.97)	**0.023**.	0.90 (0.61–1.35)	0.639	0.87 (0.76–1.00)	0.051
Age of the household head (years)						
15–29	Ref		Ref		Ref	
30–49	0.83 (0.7–0.99)	**0.046**	0.92 (0.59–1.41)	0.708	0.84 (0.72–0.98)	**0.027**
Above 50	0.76 (0.62–0.93)	**0.010**	1.06 (0.65–1.73)	0.813	0.99 (0.83–1.18)	0.963
Household wealth						
Poorest	Ref		Ref		Ref	
Poorer	0.87 (0.71–1.05)	0.166	0.54 (0.31–0.94)	**0.031**	0.91 (0.76–1.09)	0.330
Middle	0.71 (0.58–0.87)	**0.001**	1.12 (0.70–1.79)	0.633	0.91 (0.76–1.09)	0.356
Richer	0.50 (0.40–0.63)	**< 0.001**	0.87 (0.50–1.51)	0.635	0.89 (0.73–1.09)	0.278
Richest	0.26 (0.20–0.36)	**< 0.001**	1.02 (0.54–1.93)	0.931	0.86 (0.69–1.09)	0.228
Health insurance coverage						
No	Ref		Ref		Ref	
Yes	0.68 (0.44–1.04)	0.079	0.13 (0.02–1.00)	0.050	0.53 (0.39–0.71)	**< 0.001**
Water sources						
Protected	Ref		Ref		Ref	
Unprotected	0.95 (0.81–1.11)	0.557	1.08 (0.73–1.59)	0.684	0.96 (0.84–1.11)	0.651
Presence of mosquito net						
No	Ref		Ref		Ref	
Yes	0.79 (0.67–0.94)	**0.009**	1.07 (0.79–1.66)	0.733	0.92 (0.79–1.06)	0.271

*Note:* The bold value indicates the significant factors as of *p* < 0.05.

Abbreviations: AOR&amp;#x02009;=&amp;#x02009;adjusted odds ratio; CI&amp;#x02009;=&amp;#x02009;confidence interval; Ref&amp;#x02009;=&amp;#x02009;reference category.

### 3.4 Maternal Factors on Severity of Undernutrition

Table 4 shows the odds ratio from multivariable ordinal logistic regression for the association between maternal factors and severity of malnutrition. Maternal nutrition status, current age, age at first birth, education, marital status, and occupation were associated with severity of undernutrition in Tanzania. Children from normal‐weight and overweight mothers were less likely to be wasted compared to underweight mothers (AOR = 0.29, 95% CI; 0.17–0.51). However, children from normal‐weight mothers were more likely to be stunted (AOR = 1.71, 95% CI; 1.20–2.44) than underweight mothers. Furthermore, the likelihood of severity of anemia decreases as the age of mothers increases from a younger age range of 15–24 years to an older age range of 25–39 years (AOR = 0.58, 95% CI; 0.43–0.78). Mothers engaged in farming activities had an increased likelihood of severe stunting compared to mothers who were not employed (AOR = 1.39, 95% CI; 1.11–1.75). In this study, higher educational attainment lowers the severity of stunting and anemia. Severity of childhood anemia was associated with the anemic status of mothers.

**TABLE 4:** Ordinal logistic regression analysis between maternal factors on the severity of malnutrition among children aged 6–59 months in Tanzania.

Variables	Stunting		Wasting		Anemia	
	AOR (95% CI)	*p*	AOR (95% CI)	*p*	AOR (95% CI)	*p*
Nutrition status of mother (BMI)						
Underweight	Ref		Ref		Ref	
Normal	1.71 (1.20–2.44)	**0.003**	0.29 (0.17–0.51)	**< 0.001**	0.78 (0.58–1.04)	0.101
Overweight	1.24 (0.82–1.86)	0.298	0.29 (0.14–0.62)	**0.001**	0.62 (0.45–0.87)	**0.005**
Obesity	1.00 (0.63–1.58)	0.992	0.52 (0.25–1.11)	0.095	0.73 (0.51–1.05)	0.099
Mother's age (years)						
15–24	Ref		Ref		Ref	
25–39	1.00 (0.82–1.22)	0.937	1.41 (0.85–2.35)	0.179	0.77 (0.64–0.91)	0.003
40–49	0.92 (0.66–1.30)	0.667	0.86 (0.34–2.13)	0.748	0.58 (0.43–0.78)	**< 0.001**
Mother's age at first birth (years)	0.98 (0.80–1.21)	0.914	1.83 (1.16–2.88)	**0.008**	0.95 (0.79–1.13)	0.571
Number of births in the last 3 years	1.37 (1.10–1.71)	**0.005**	0.93 (0.53–1.64)	0.819	1.00 (0.82–1.22)	0.947
Mother's occupation						
Not working	Ref		Ref		Ref	
Farmer	1.39 (1.11–1.75)	**0.004**	0.97 (0.56–1.68)	0.938	0.81 (0.66–1.00)	0.054
Employed	0.99 (0.80–1.22)	0.953	0.65 (0.39–1.07)	0.092	0.90 (0.76–1.08)	0.282
Mother's education						0.155
No education	Ref		Ref		Ref	
Primary	0.79 (0.65–098)	**0.035**	0.63 (0.37–1.07)	0.090	0.77 (0.63–0.94)	**0.013**
Secondary	0.56 (0.42–0.74)	**< 0.001**	0.79 (0.43–1.47)	0.467	0.79 (0.62–1.00)	0.059
Higher	0.19 (0.04–0.85)	**0.030**	NA	0.990	1.27 (0.55–2.94)	0.567
Place of delivery						
At health facility	Ref		Ref		Ref	
At home	1.02 (0.81–1.28)	0.845	1.26 (0.74–2.12)	0.386	1.09 (0.89–1.33)	0.393
Other places						
Number of antenatal care visits						
No	Ref		Ref		Ref	
1–5	0.86 (0.64–1.14)	0.308	1.64 (0.76–3.57)	0.205	1.08 (0.84–1.39)	0.538
≥ 6	0.87 (0.63–1.19)	0.406	1.29 (0.54–3.04)	0.560	0.92 (0.69–1.22)	0.582
Mother's anemia status						
No	Ref		Ref		Ref	
Mild	0.94 (0.75–1.17)	0.617	1.00 (0.58–1.70)	0.998	1.39 (1.15–1.68)	**< 0.001**
Moderate	1.06 (0.85–1.34)	0.562	1.24 (0.73–2.10)	0.419	1.73 (1.41–2.13)	**< 0.001**
Severe	0.91 (0.49–1.67)	0.763	1.17 (0.34–4.01)	0.794	1.78 (1.05–3.02)	**< 0.001**
Current marital status						
No	Ref		Ref		Ref	
Yes	0.77 (0.61–0.77)	**0.031**	0.96 (0.53–1.73)	0.894	0.87 (0.70–1.07)	0.198

Abbreviations: AOR = adjusted odds ratio; CI = confidence interval; Ref = reference category.

## 4 Discussion

By using nationally representative data from Tanzania, this study tried to ascertain the prevalence and associated factors of severe undernutrition. The prevalences of severe stunting, wasting, and anemia among Tanzanian children were 8.6%, 0.8%, and 1.7%, respectively. The prevalence of multiple forms of severe undernutrition was 10.4%. The high prevalence of multiple forms of severe undernutrition indicates the existence of a double‐burden of severe acute and chronic undernutrition and micronutrient deficiency. This highlights that one in every 10 under‐five children is suffering from severe undernutrition in Tanzania. This finding is similar to reported studies in Tanzania and other countries (Rasheed and Jeyakumar 2018; Khamis et al. 2020; Belay et al. 2023; Pradeilles et al. 2022).

Furthermore, this study shows that the severity levels of stunting, wasting, and anemia were determined by maternal, socioeconomic, and dietary factors. These findings show that Tanzanian children are at the center of various stresses that impact their potential for good growth and development (Sunguya et al. 2019). In this study, five socioeconomic variables were found as major determinants of severe levels of undernutrition: the household head's gender, age, wealth position, health insurance coverage, and the existence of a mosquito net. Overall, the study reveals the complex interplay of factors that contribute to severe undernutrition in Tanzania, implying that combating undernutrition may require a diverse approach to address many kinds of malnutrition in Tanzania (Khamis et al. 2020).

These findings align with findings from earlier studies that explored the prevalence and determinants of severe undernutrition (Ahmad, Afzal, and Imtiaz 2020; Chowdhury et al. 2023). The study indicates that children residing in rural areas face a greater risk of severe undernutrition compared to their urban counterparts, consistent with earlier studies undertaken in Ethiopia (Alemayehu, Cherie, and Chernet 2022; Muchie 2016) and Pakistan (Siddiqa et al. 2022), and this may be attributed to better living conditions and food security in urban areas. This study shows that children from economically disadvantaged households are more likely to experience severe undernutrition than those from a wealthier background, echoing numerous studies conducted previously in developing countries like Ethiopia (Endris, Asefa, and Dube 2017), Bangladesh (Islam and Biswas 2019), and India (Dasgupta et al. 2014). In comparison, a study conducted in Sub‐Saharan Africa demonstrated that the severity of anemia correlated significantly with socioeconomic factors, including maternal education, household wealth status, and family size (Tesema et al. 2021).

This study identified six other maternal factors that contribute to the severity of undernutrition among young children in Tanzania: the nutritional status of the mother and her current age, mother's age at first birth, occupation, education, and marital status. Hence, maternal factors are very important to consider when addressing the problems associated with severe undernutrition in Tanzania. For instance, maternal anemia was significantly associated with higher odds of anemia severity among children, consistent with studies conducted in Sub‐Saharan African (Tesema et al. 2021) and Asian countries (Khan, Awan, and Misu 2016). This association may be because mothers are the primary source who provide food to children; consequently, their eating habits can be similar to those of her children (Baughcum et al. 1998). In addition, factors such as placental transmission, breastfeeding, and infectious causes of anemia can sometimes impact the development of red blood cells and iron stores, which may affect infants. Also, anemic mothers may lack enough essential nutrients such as iron, zinc, and folate in their breast milk, potentially contributing to childhood anemia (Baker and Greer 2010). This calls for additional efforts focusing on maternal and child health.

The findings from this study affirm earlier studies in Tanzania that have documented the role of improved household wealth status in the prevention of the severity of stunting and wasting. A previous study implemented in Tanzania to assess the drivers of malnutrition has confirmed that income poverty is a major barrier to preventing access to nutritious complementary foods (Kulwa et al. 2015). On the other hand, female‐headed households were less likely to have severe stunting than male‐headed ones. This observation is confirmed by a recent study in Tanzania, which has shown that women decide on what to purchase and cook for the family depending on the amount of money, as well as decide on the number of meals per day if food is available. Unlike married women, men dominate much of the household's decision making, especially on matters related to money or purchases, implying that a child cared for by them stands to be nutritionally best, provided that other factors remain constant. In this study, the severity of stunting and anemia was low among older mothers aged 30–49 years compared to the younger category of 15–29 years. While it is crucial to consider the age of the mother when addressing issues of nutrition, it is notable that the median age at first live birth among women aged 25–49 years is 20 and 19.5, respectively, indicating that the majority of the caregivers belong to the lowest age bracket, thus signifying a potential risk for malnutrition for children cared for by these mothers. In Tanzania, owning a health insurance coverage scheme signifies access to established health services within the country. To greater extents, employees in formal and private sectors enjoy this noble privilege compared to the large majority of the Tanzanian population. While the WHO advocates for universal health coverage to all, which is also supported by the Tanzanian government, more needs to be done to attain this goal. Based on the 2023 demographic and health survey data, only 59% of the children under the age of five slept under insecticide‐treated nets on the night preceding the survey. Sleeping under a mosquito net not only is an indication of a good care practice for the child but also provides protection against malaria.

Overall, this study nuanced the identification of socioeconomic and maternal factors contributing to severe undernutrition in Tanzania. The study highlights not only the prevalence of multiple forms of undernutrition but also the significant role that maternal factors, such as nutritional status, age, and occupation, play in a child's health. Additionally, the study observed that female‐headed households may better prevent severe stunting since decision‐making around nutrition is an important insight. This suggests that empowering women in household decisions and improving maternal health can have a profound impact on reducing severe undernutrition. This multidimensional approach may signal a shift from focusing only on child health to addressing broader socioeconomic and gender‐related determinants in combating undernutrition.

This study has some limitations. Analysis of the prevalence and determinants of undernutrition were only identified from cross‐sectional data, and it is possible that this could have no causal relationship among variables. Also, this study did not incorporate other potential factors that could contribute to undernutrition, such as data on water hygiene and sanitation. It is crucial to include these factors in future research endeavors. However, this study has some strengths. We anticipate that it will illuminate critical areas for undernutrition interventions in Tanzania. Moreover, the study utilized large and nationally representative data with sufficient sample size at both national and subnational levels, offering the most reliable estimates for the entire country.

## 5 Conclusion

In Tanzania, one out of every 10 children under the age of five is experiencing different forms of severe undernutrition. Multidimensional factors, particularly those related to maternal health and household socioeconomic status, have the ability to minimize the severity of childhood undernutrition. Therefore, efforts to alleviate the severity of undernutrition should focus on addressing poverty and maternal and child health factors.

## Author Contributions

**Ahmed Gharib Khamis:** conceptualization (equal), data curation (equal), formal analysis (equal), investigation (equal), methodology (equal), resources (equal), software (equal), validation (equal), writing – original draft (equal), writing – review and editing (equal). **Akwilina Wendelin Mwanri:** conceptualization (equal), methodology (equal), project administration (equal), supervision (equal), writing – original draft (equal), writing – review and editing (equal). **Julius Edward Ntwenya:** conceptualization (equal), methodology (equal), writing – original draft (equal), writing – review and editing (equal).

## Ethics Statement

This study was approved by the relevant authorities in Tanzania. The ethical approvals were from the National Institute of Medical Research (NIMR) and the Zanzibar Health Research Institute (ZAHRI). Also, this was authorized by the Institutional Review Board (IRB) such as ICF and the Centers for Disease Control (CDC) (Ministry of Health CD 2018). In this study, consent was not necessary and no personal identification was extracted from the database.

## Consent

The authors have nothing to report.

## Conflicts of Interest

The authors declare no conflicts of interest.

## Data Availability

All data for this study are available for free on request from the Demographic and Health Survey (DHS) website of www.dhsprogram.com.

## References

[fsn34648-bib-0001] Ahmad, D. , M. Afzal , and A. Imtiaz . 2020. “Effect of Socioeconomic Factors on Malnutrition Among Children in Pakistan.” Future Business Journal 6, no. 1: 30.

[fsn34648-bib-0002] Ahmad, I. , N. Khalique , and S. Khalil . 2018. “Dietary Diversity and Stunting Among Infants and Young Children: A Cross‐Sectional Study in Aligarh.” Indian Journal of Community Medicine: Official Publication of Indian Association of Preventive & Social Medicine 43, no. 1: 34–36. 29531436 10.4103/ijcm.IJCM_382_16 PMC5842471

[fsn34648-bib-0003] Alemayehu, G. M. , K. T. Cherie , and A. G. Chernet . 2022. “Severity of Malnutrition Among Underweight Children and Family‐Related Factors: A Cross‐Sectional Analysis of Data From the 2019 Ethiopian Demographic and Health Survey (EDHS).” Health Science Reports 5, no. 6: e860. 36210870 10.1002/hsr2.860 PMC9528949

[fsn34648-bib-0004] Arimond, M. , and M. T. Ruel . 2004. “Dietary Diversity Is Associated With Child Nutritional Status: Evidence From 11 Demographic and Health Surveys.” Journal of Nutrition 134, no. 10: 2579–2585. 15465751 10.1093/jn/134.10.2579

[fsn34648-bib-0005] Baker, R. D. , and F. R. Greer . 2010. “Nutrition TCo: Diagnosis and Prevention of Iron Deficiency and Iron‐Deficiency Anemia in Infants and Young Children (0–3 Years of Age).” Pediatrics 126, no. 5: 1040–1050. 20923825 10.1542/peds.2010-2576

[fsn34648-bib-0006] Baughcum, A. E. , K. A. Burklow , C. M. Deeks , S. W. Powers , and R. C. Whitaker . 1998. “Maternal Feeding Practices and Childhood Obesity: A Focus Group Study of Low‐Income Mothers.” Archives of Pediatrics & Adolescent Medicine 152, no. 10: 1010–1014. 9790612 10.1001/archpedi.152.10.1010

[fsn34648-bib-0007] Beckstead, E. , G. Mulokozi , M. Jensen , et al. 2022. “Addressing Child Undernutrition in Tanzania With the ASTUTE Program.” BMC Nutrition 8, no. 1: 29. 35392969 10.1186/s40795-022-00511-0 PMC8988343

[fsn34648-bib-0008] Belay, D. G. , D. Chilot , A. Z. Alem , F. M. Aragaw , and M. H. Asratie . 2023. “Spatial Distribution and Associated Factors of Severe Malnutrition Among Under‐Five Children in Ethiopia: Further Analysis of 2019 Mini EDHS.” BMC Public Health 23, no. 1: 791. 37118793 10.1186/s12889-023-15639-2 PMC10142160

[fsn34648-bib-0009] Chirande, L. , D. Charwe , H. Ally , et al. 2015. “Determinants of Stunting and Severe Stunting Among Under‐Fives in Tanzania: Evidence From the 2010 Cross‐Sectional Household Survey.” BMC Pediatrics 15: 165. 26489405 10.1186/s12887-015-0482-9 PMC4618754

[fsn34648-bib-0010] Chowdhury, M. R. K. , M. S. Rahman , B. Billah , M. Rashid , M. Almroth , and M. Kader . 2023. “Prevalence and Factors Associated With Severe Undernutrition Among Under‐5 Children in Bangladesh, Pakistan, and Nepal: A Comparative Study Using Multilevel Analysis.” Scientific Reports 13, no. 1: 10183. 37349482 10.1038/s41598-023-36048-w PMC10287716

[fsn34648-bib-0011] Das, S. , and R. M. Rahman . 2011. “Application of Ordinal Logistic Regression Analysis in Determining Risk Factors of Child Malnutrition in Bangladesh.” Nutrition Journal 10: 124. 22082256 10.1186/1475-2891-10-124 PMC3296641

[fsn34648-bib-0012] Dasgupta, P. A. , R. Parthasarathi , V. Prabhakar , R. Biswas , and A. Geethanjali . 2014. “Assessment of Under Nutrition With Composite Index of Anthropometric Failure (CIAF) Among Under‐Five Children in a Rural Area of West Bengal.” Indian Journal of Community Health 26: 132–138.

[fsn34648-bib-0013] De Sanctis, V. , A. Soliman , N. Alaaraj , S. Ahmed , F. Alyafei , and N. Hamed . 2021. “Early and Long‐Term Consequences of Nutritional Stunting: From Childhood to Adulthood.” Acta Bio‐Medica: Atenei Parmensis 92, no. 1: e2021168. 33682846 10.23750/abm.v92i1.11346 PMC7975963

[fsn34648-bib-0014] Endris, N. , H. Asefa , and L. Dube . 2017. “Prevalence of Malnutrition and Associated Factors Among Children in Rural Ethiopia.” BioMed Research International 2017: 1–6. 10.1155/2017/6587853 PMC5449753 28596966

[fsn34648-bib-0015] Govender, I. , S. Rangiah , R. Kaswa , and D. Nzaumvila . 2021. “Malnutrition in Children Under the Age of 5 Years in a Primary Health Care Setting.” South African Family Practice: Official Journal of the South African Academy of Family Practice/Primary Care 63, no. 1: e1–e6. 10.4102/safp.v63i1.5337 PMC8517826 34677078

[fsn34648-bib-0016] Islam, M. S. , and T. Biswas . 2019. “Prevalence and Correlates of the Composite Index of Anthropometric Failure Among Children Under 5 Years Old in Bangladesh.” Maternal & Child Nutrition 16: e12930. 31867876 10.1111/mcn.12930 PMC7083426

[fsn34648-bib-0017] Khamis, A. G. , A. W. Mwanri , K. Kreppel , and G. Kwesigabo . 2020. “The Burden and Correlates of Childhood Undernutrition in Tanzania According to Composite Index of Anthropometric Failure.” BMC Nutrition 6, no. 1: 39.

[fsn34648-bib-0018] Khamis, A. G. , A. W. Mwanri , J. E. Ntwenya , and K. Kreppel . 2019. “The Influence of Dietary Diversity on the Nutritional Status of Children Between 6 and 23 Months of Age in Tanzania.” BMC Pediatrics 19, no. 1: 518. 31881999 10.1186/s12887-019-1897-5 PMC6935228

[fsn34648-bib-0019] Khan, J. R. , N. Awan , and F. Misu . 2016. “Determinants of Anemia Among 6–59 Months Aged Children in Bangladesh: Evidence From Nationally Representative Data.” BMC Pediatrics 16: 3. 26754288 10.1186/s12887-015-0536-z PMC4707771

[fsn34648-bib-0020] Kulwa, K. B. M. , P. S. Mamiro , M. E. Kimanya , R. Mziray , and P. W. Kolsteren . 2015. “Feeding Practices and Nutrient Content of Complementary Meals in Rural Central Tanzania: Implications for Dietary Adequacy and Nutritional Status.” BMC Pediatrics 15, no. 1: 171. 26546052 10.1186/s12887-015-0489-2 PMC4636743

[fsn34648-bib-0021] Ministry of Health CD, Gender and Elderly, Ministry of Health (Zanzibar), National Bureau of Statistics, OCGS, and ICF . 2015. “Tanzania Demographic and Health Survey Malaria Indicator Survey (TDHS‐MIS).”

[fsn34648-bib-0022] Ministry of Health CD, Gender, Elderly and Children (MoHCDGEC) [Tanzania Mainland], Ministry of Health (MoH) [Zanzibar], Tanzania Food and Nutrition Centre (TFNC), National Bureau of Statistics (NBS), Office of the Chief Government Statistician (OCGS) [Zanzibar] and UNICEF . 2018. “Tanzania National Nutrition Survey Using SMART Methodology (TNNS).” In Dar es Salaam, Tanzania.

[fsn34648-bib-0023] Ministry of Health, Community Development, Gender, Elderly and Children (MoHCDGEC) [Tanzania Mainland], Ministry of Health (MoH) [Zanzibar], National Bureau of Statistics(NBS), Office of the Chief Government Statistician (OCGS), and ICF . 2016. “Tanzania Demographic and Health Survey and Malaria Indicator Survey (TDHS‐MIS).” 2015–16. Dar‐es‐Salaam, Tanzania, and Rockville, Maryland, USA: MoHCDGEC, MoH, NBS, OCGS, and ICF.

[fsn34648-bib-0024] Motbainor, A. , A. Worku , and A. Kumie . 2015. “Stunting Is Associated With Food Diversity While Wasting With Food Insecurity Among Underfive Children in East and West Gojjam Zones of Amhara Region, Ethiopia.” PLoS One 10, no. 8: e0133542. 26285047 10.1371/journal.pone.0133542 PMC4540277

[fsn34648-bib-0025] Muchie, K. F. 2016. “Determinants of Severity Levels of Anemia Among Children Aged 6–59 Months in Ethiopia: Further Analysis of the 2011 Ethiopian Demographic and Health Survey.” BMC Nutrition 2, no. 1: 51.

[fsn34648-bib-0026] Obasohan, P. E. , S. J. Walters , R. Jacques , and K. Khatab . 2020. “Risk Factors Associated With Malnutrition Among Children Under‐Five Years in Sub‐Saharan African Countries: A Scoping Review.” International Journal of Environmental Research and Public Health 17, no. 23: 8782. 33256022 10.3390/ijerph17238782 PMC7731119

[fsn34648-bib-0027] Pradeilles, R. , A. Irache , T. Norris , S. Chitekwe , A. Laillou , and K. Baye . 2022. “Magnitude, Trends and Drivers of the Coexistence of Maternal Overweight/Obesity and Childhood Undernutrition in Ethiopia: Evidence From Demographic and Health Surveys (2005–2016).” Maternal & Child Nutrition 20: e13372. 35615766 10.1111/mcn.13372 PMC11258774

[fsn34648-bib-0028] Rasheed, W. , and A. Jeyakumar . 2018. “Magnitude and Severity of Anthropometric Failure Among Children Under Two Years Using Composite Index of Anthropometric Failure (CIAF) and WHO Standards.” International Journal of Pediatrics and Adolescent Medicine 5, no. 1: 24–27. 30805528 10.1016/j.ijpam.2017.12.003 PMC6363267

[fsn34648-bib-0029] Siddiqa, M. , A. Zubair , A. Kamal , M. Ijaz , and T. Abushal . 2022. “Prevalence and Associated Factors of Stunting, Wasting and Underweight of Children Below Five Using Quintile Regression Analysis (PDHS 2017–2018).” Scientific Reports 12, no. 1: 20326. 36434025 10.1038/s41598-022-24063-2 PMC9700674

[fsn34648-bib-0030] Sunguya, B. F. , S. Zhu , R. Mpembeni , and J. Huang . 2019. “Trends in Prevalence and Determinants of Stunting in Tanzania: An Analysis of Tanzania Demographic Health Surveys (1991–2016).” Nutrition Journal 18, no. 1: 85. 31823827 10.1186/s12937-019-0505-8 PMC6904996

[fsn34648-bib-0031] Suryawan, A. , M. Y. Jalaludin , B. K. Poh , et al. 2022. “Malnutrition in Early Life and Its Neurodevelopmental and Cognitive Consequences: A Scoping Review.” Nutrition Research Reviews 35, no. 1: 136–149. 34100353 10.1017/S0954422421000159

[fsn34648-bib-0032] Tesema, G. A. , M. G. Worku , Z. T. Tessema , et al. 2021. “Prevalence and Determinants of Severity Levels of Anemia Among Children Aged 6–59 Months in Sub‐Saharan Africa: A Multilevel Ordinal Logistic Regression Analysis.” PLoS One 16, no. 4: e0249978. 33891603 10.1371/journal.pone.0249978 PMC8064743

[fsn34648-bib-0033] WHO . “Physical Status and the Use and Interpretation of Anthropometry.” Reports of WHO Expert Committee, Technical Report Series, 854, Geneva, Switzerland. 13–125 (2006). 8594834

[fsn34648-bib-0034] World Bank . n.d. “Tanzania's Path to Poverty Reduction and Pro‐Poor Growth.” https://www.worldbank.org/en/country/tanzania/publication/tanzanias‐path‐to‐poverty‐reduction‐and‐pro‐poor‐growth.

[fsn34648-bib-0035] World Health Organization . 2022. “Malnutrition Fact Sheet.” https://www.who.int/news‐room/fact‐sheets/detail/malnutrition.

[fsn34648-bib-0036] World Health Organization . n.d. “Infant and Young Child Feeding.” https://www.who.int/news‐room/fact‐sheets/detail/infant‐and‐young‐child‐feeding.

